# What Is the Right Mechanical Readout for Understanding the Mechanobiology of the Immune Response?

**DOI:** 10.3389/fcell.2021.612539

**Published:** 2021-02-25

**Authors:** Marco Fritzsche

**Affiliations:** ^1^Rosalind Franklin Institute, Didcot, United Kingdom; ^2^Kennedy Institute for Rheumatology, University of Oxford, Oxford, United Kingdom

**Keywords:** mechanics, biophysics, mechanical force, mechanical properties, feedback, dynamics, stiffness, tension

Mechanobiology is a critical frontier in the biomedical sciences. Across many of its fields a new perspective is emerging to perceive the immune response as a single multi-scale super-organism continuously interacting and interpreting the biochemical and biomechanical micro-environment. Large numbers of immune cells communicate through a combination of chemical and mechanical signals to organise and orchestrate their behaviour and function against an immunological threat. However, disease often circumvents and even exploits mechanobiological features of this defence machinery, highlighting the need to better understand the intimate coupling between biology and mechanics.

Mechanical force underpins the immune response at the multi-scale (Fritzsche, 2020). While almost all physical forces relevant to immune cell biology are practically restricted to the sub-cellular level such as electrostatics (e.g., receptor ligand binding) and thermodynamics (e.g., molecule diffusion), biomechanics takes on a special importance as mechanical force influences many functional features and behavior of immune cells over multiple scales in space and time (Dumont and Prakash, 2014; Egan et al., 2015). Mechanics contributes to the dynamics of single molecules, cells, tissues, and entire organisms (Blanchard and Adams, 2011; Chen and Zhu, 2013). The effects on the biology result from contributions of mechanics that are combination of those generated locally and those that influence from a distance. Importantly, both mechanical force and mechanical properties differ at distinct spatio-temporal frequencies such as constant and oscillatory forces, tension, or elasticity and viscosity (see Figure 1), respectively. For example, living cells can be elastic, viscous, or visco-elastic depending on the spatial and temporal measurement frequency (see Figure 1A). Adequate quantification promises thus a deeper understanding of the multi-scale spatio-temporal coupling of biology and mechanics and its control over the immune response (Fritzsche, 2020).

**Figure 1:**
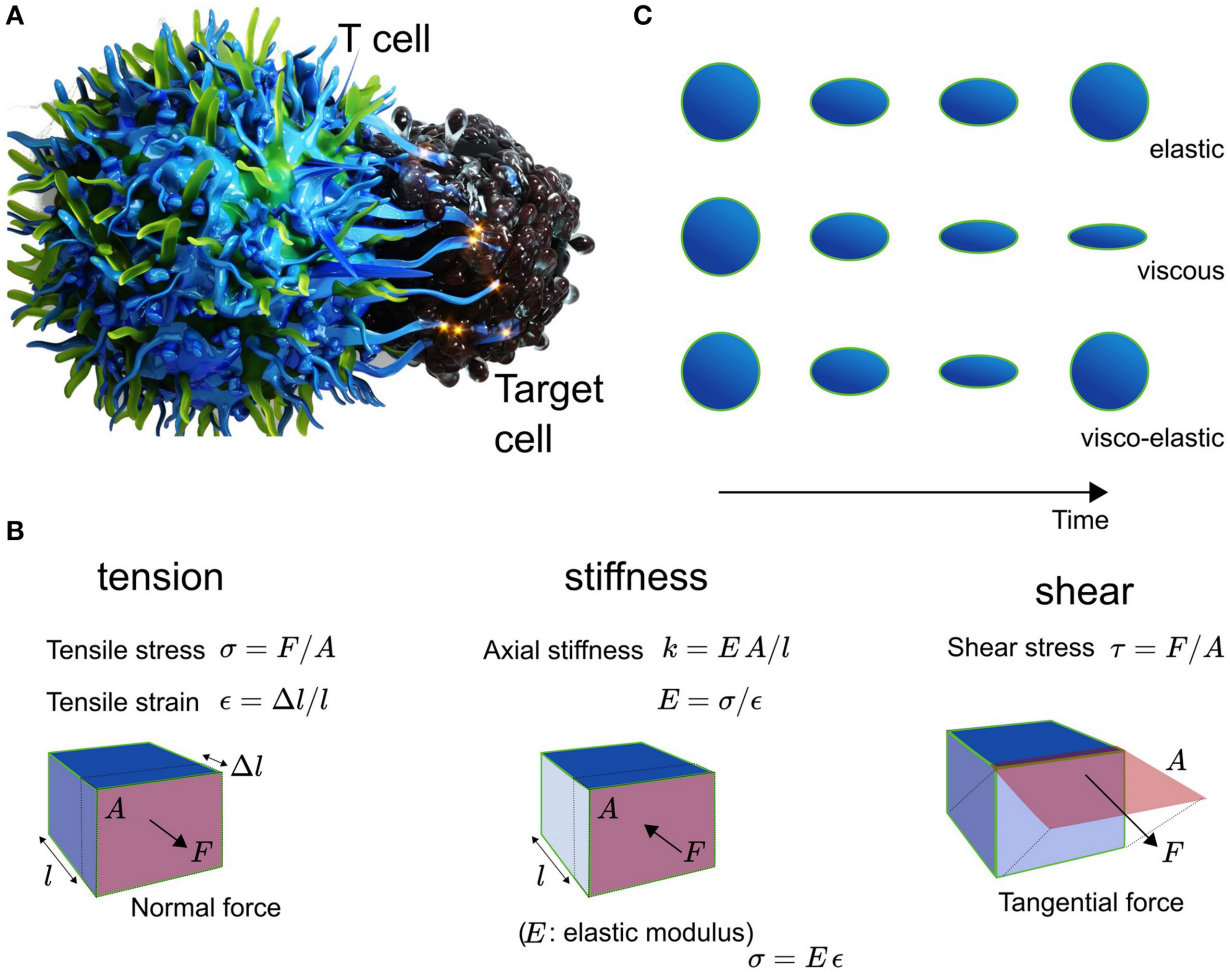
**(A)** Schematic of the T cell (left) and Target cell (right) interplay. **(B)** Explanation of the mechanical metric tension with tensile stress σ in units of N/m^2^ and tensile strain ε being dimenionless, and shear with shear stress τ in N/m^2^, as well as the elasticity with Youngst modulus E, which equals the ratio of stress over strain. The stiffness of a material is given by EA/l. **(C)** Cells are elastic, viscous, or visco-elastic.

Central to the immune defense against an invading threat is a well-orchestrated sequence of events carried out by specialized cells of the multi-scale super-organism of the immune system (Chaplin, 2010). The Success of the process relies on the quality of the spatiotemporal organization of the cellular responses in the tissue micro-environment of the host organism for instance against cancer or an invading pathogenic threat (Swartz and Lund, 2012). Cytotoxic immune cells circulate through tissue, track soluble cytokines and chemokines, respond to antigens, and kill diverse immuno-targets (Andersen et al., 2006; Grivennikov et al., 2010). Among many of these events, they involve a combination of biological and mechanical spheres of influence, as immune cells continuously interact and interpret the physical surroundings (Colin-York et al., 2016; Schwarz, 2017). This environment is also mechanically diverse over scales of space and time. It is comprised of different types of molecules, cells, and tissues (Needleman and Dogic, 2017). Mechanical aspects of the tissue environment are known to influence both the function and behavior of cells (Schwarz, 2017; Colin-York and Fritzsche, 2018). The complex multi-scale nature of mechanical force may have evolutionary been the reason for immune cells to develop the ability to adjust their own biomechanics to their physiological needs in response to the ever-changing physical world. Strikingly, when cells loose mechanosensation, the immune response is hampered to robustly and/or reliably achieve its protective function, which has been demonstrated for example during cell-cell interactions such as activation and cytotoxicity (Huse, 2017; Kumari et al., 2019), as well as during cellular interactions with the tumor micro-environment (Mohammadi and Sahai, 2018; Majedi et al., 2020). Consequently, this gives a special significance to mechanobiology of being important for the understanding of the functioning of the immune response as a whole in health and disease.

One of the most illustrative and visually impressive examples for the impact of mechanobiology is the activation of T cells and antigen presenting cells (APCs) (Chen and Zhu, 2013; Harrison et al., 2019). Micron-scale ruffles protruding from the T cell initiate contact and binding between T cell receptors (TCRs) and the APC's peptide-loaded major hist-compatibility complexes (pMHCs) (Fritz-Laylin et al., 2017; Fritzsche et al., 2017). In the event of recognition and binding of pMHCs by a TCR, the T cell rearranges, assisted by its cytoskeleton, the totality of its metabolism, inner organelles, membrane, and its surface receptors and ligands (English and Voeltz, 2013; Maciver et al., 2013; Carlton et al., 2020). Strikingly, physical symmetry plays a major factor in these re-arrangements, possibly because of the need to balance and direct mechanical force between the T cell and the APC (Sims et al., 2007; Dustin, 2009; Arsenio et al., 2015). Calcium release in response to TCR-pMHC binding leads to the depolymerisation of microtubules, which in turn facilitates the rearrangement of organelles such as the nucleus and endoplasmic reticulum to the center of the T-cell body volume (Joseph et al., 2014; Ilan-Ber and Ilan, 2019). Cytoskeletal ruffles depolymerise and actin-rich lamellum and lamellipodium polymerise at the contact between both cells. The lamellipodium constantly propels freshly forming TCR clusters to the center of the contact (Fritzsche et al., 2017), whose function is thought to amplify the pMHC binding and recognition (Harrison et al., 2019). At the interface between the T cell and the APC, the immunological synapse (IS) takes shape involving a complex spatio-temporal orchestration of receptors, positive and negative co-stimulatory co-receptors, and integrins (Dustin, 2009). They contribute in concert to the force balance between the T cell and the APC. As antigens are processed at the IS, the T cell ensures a mechanically stable and flat contact interface in the form of a ramified actin network (Fritzsche et al., 2017). Visual inspection suggested, that this network is shaped under mechanical tension and held stable over time through the actively polymerising actin-rich lamellipodium and its shear force producing actin retrograde flow (Colin-York et al., 2019b). These interfacial processes are further supported by mechanically active protrusions (Tamzalit et al., 2019). On one side of IS, the T cell constantly assembles and disassembles short-lived actin foci facilitating and perhaps ensuring localized contact between both cells (Kumari et al., 2019, 2020). On the other side of the interaction, the APC forms mechano-transducing podosomes in the outer periphery of the IS (Malinova et al., 2016), which may serve to monitor the mechanical stiffness at the contact interface. The mechanical properties of the cytoskeletal actin architectures and protrusions are also time-dependent with distinct viscosities depending on the observation frequency, because they and their crosslinkers are constantly turning over (Fritzsche et al., 2016; Gat et al., 2020). Loss of symmetry at the IS, and thus loss of mechanical force balance at the contact, has been demonstrated to influence instability and success of immune cell activation (Sims et al., 2007; Lee et al., 2017). These outlined processes are further underpinned by dynamic molecular changes in the nanoscale organization and turnover of actin filaments in the actin cortex and lamellipodium as a function of the antigen affinity (Billadeau et al., 2007; Colin-York et al., 2019b; Wahl et al., 2019), suggesting mechanical feedback on multiple length- and time-scales. Over the years, a multitude of measurements in the study of immune cell activation reported the importance of different mechanical metrics such as stiffness, tension, shear, and structural integrity, highlighting the diversity of current mechanical quantifications (Figure 1B) (Egan et al., 2015).

Together, having uncovered the presence of a variety of length- and time-scale dependent mechanical force regimes and properties during immune cell activation (Pageon et al., 2018), and moreover, knowing that T cells and APCs integrate a variety of mechanical readouts (Jain et al., 2019), leads to the question what mechanical measurements are necessary and sufficient to understand the mechanobiology of the immune response? More specifically, how do these mechanical signatures couple and feed into biology over space and time, and are thus integrated into cellular function and behavior (Harris et al., 2018)? To add further complexity to this picture, the T cell-APC interplay is usually not insulated but maintains processes of continuous communication and interpretation of the surrounding biochemical and biomechanical micro-environment. Consequently, without understanding comprehensively the dynamic relationship of these processes, it is challenging to determine the biological significance of mechanobiology in health and disease.

Hence, a grand challenge for the understanding of mechanbiology is the determination of the right mechanical readout. Ideally, from the theoretical physics point of view, one aims for a full quantitative parametrisation of the desired biological phenomena, which ultimately comes down to identification and determination of a well-defined control parameter (Bechhoefer, 2005). The determinant at which biomechanics regulates behavior and/or function of the immunological process of interest. The prospect of knowing the one (or the many) mechanical control parameter(s) against all other system parameters is of particular importance in the context of stability of the biological function (Bechhoefer, 2005; McEvoy, 2018). For example the stability of IS formation could be regulated through changes in mechanical feedback between the T cell and the APC (Harrison et al., 2019). Stability of such a biological feature is mathematically determined by the so-called eigenmodes of its stability matrix, which grow or shrink when the control parameter for example mechanical feedback changes (Bechhoefer, 2005). Crucially, while experimentally, changes in different mechanical parameters could be observed throughout a biological process (Dumont and Prakash, 2014; Egan et al., 2015), only the determination of the biological control parameter aids to the understanding of the biological mechanisms and concepts being at play (McEvoy, 2018). In other words, one may observe quantitative changes in mechanical metrics such as stiffness and/or tension but in practice misinterpret the observed phenomena, let it be for example mechanical feedback during IS formation, if the control parameter *feedback* has not been correctly identified and parameterized.

## Future Perspective

How to then find the right mechanical readout? The answer to this question is not trivial and practically challenging for many biological systems due to the numerous molecular players involved, the number and complexity of their interactions, but probably mostly due to a broad lack of quantitative technology with the right sensitivity (Polacheck and Chen, 2016; Roca-Cusachs et al., 2017).

Over the last 10 years, recent advances in quantitative technology have enabled the spatio-temporal sensitivity demanded by the immune response drawing a promising perspective for the future. These new methodologies will enable a complete quantitative characterization of biological processes to the best of the experimentalist's abilities allowing the full parameterization of theoretical physics descriptions (McEvoy, 2018). For this, a variety of different technologies are needed to quantify correlatively or co-incidentally the mechanical setting with the sensitivity demanded by the biology of interest. We and others have spent significant efforts in evolving the sensitivity of traction force microscopy, probably the most widely applied force quantification methodology, which offers the simultaneous quantification of mechanical force production and the dynamics of cells (Colin-York and Fritzsche, 2018; Colin-York et al., 2019a; Stubb et al., 2020; Vorselen et al., 2020). More recent efforts of combining different types of quantitative simultaneous measurements hold the promise to achieve a more complete understanding of how biomechanics feeds into immune cell physiology of the immune response (Skamrahl et al., 2019; Hobson et al., 2020; Moreno-Flores, 2020; Nelsen et al., 2020). Quantifying the mechanical settings of the immune response in full utilizing methodologies with the right sensitivity may thus be the route to enable broad recognition of mechanobiology in health and disease.

## Author Contributions

MF designed and wrote the perspective article.

## Conflict of Interest

The author declares that the research was conducted in the absence of any commercial or financial relationships that could be construed as a potential conflict of interest. The handling editor declared a past co-authorship with one of the authors MF.
